# A Front-End Circuit for Two-Wire Connected Resistive Sensors with a Wire-Resistance Compensation

**DOI:** 10.3390/s23198228

**Published:** 2023-10-03

**Authors:** Ferran Reverter

**Affiliations:** Department of Electronic Engineering, Universitat Politècnica de Catalunya—BarcelonaTech, 08860 Castelldefels, Barcelona, Spain; ferran.reverter@upc.edu; Tel.: +34-934-137076

**Keywords:** parasitic resistance, remote sensor, resistive sensor, sensor interface electronics, thermal sensor

## Abstract

In this article, a novel front-end circuit for remote two-wire resistive sensors that is insensitive to the wire resistances is proposed and experimentally characterized. The circuit relies on an OpAmp-based current source with a square-wave excitation, two twin diodes in the feedback path, and a low-pass filter at the output. Using such a circuit topology, the output is a DC voltage proportional to the sensor resistance and independent of the wire resistances. A prototype was built measuring resistances that correspond to a Pt100 thermal sensor and with different values of wire resistance. The experimental results show that the output voltage is almost insensitive to both the wire resistances and their mismatch, with a relative error (with respect to the case with null parasitic resistance) in the range of 0.01–0.03% Full-Scale Span (FSS). In addition, the proposed circuit shows a remarkable linearity (around 0.01% FSS), and again this is independent of the presence and also of the mismatch of the wire resistances.

## 1. Introduction

**Figure 1:**
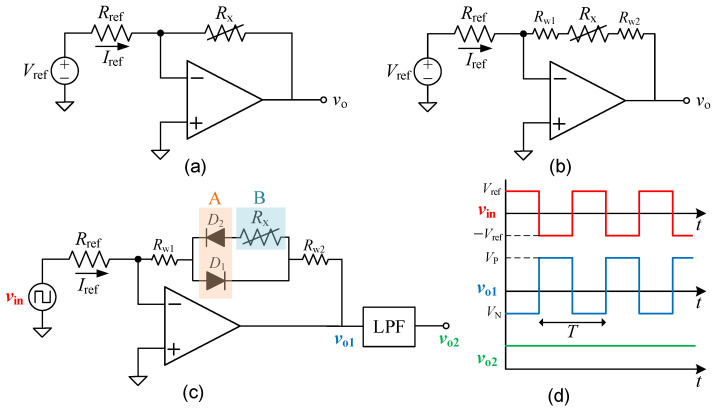
(**a**) Typical front-end circuit, acting as a constant current source, for a resistive sensor (*R*_x_). (**b**) Circuit in (**a**) affected by the parasitic resistance (*R*_w1_ and *R*_w2_) of the interconnecting wires when the resistive sensor is remote. (**c**) Proposed front-end circuit for a two-wire resistive sensor insensitive to the parasitic resistance of the wires. (**d**) Waveform of the voltage at the main nodes in the circuit shown in (**c**).

Measurement systems based on resistive sensors [1] are very common in industry and laboratory applications to measure temperature, mechanical stress, linear and angular displacement, light intensity, and gas concentration, among others. Figure 1a shows a typical front-end circuit, based on an inverting-amplifier topology, for a resistive sensor (*R*_x_) excited by a constant current (*I*_ref_) that is equal to *V*_ref_/*R*_ref_ assuming an ideal operational amplifier (OpAmp). This type of excitation applied to resistive sensors ensures a linear output signal [2] and also enables better control of the self-heating effects [3], which are especially critical in thermal resistive sensors.

Remote resistive sensors (i.e., sensors that are located at a certain distance from the read-out circuit) are quite typical in industry applications, especially when the sensor is in a harsh environment with extreme operating conditions, for example, when the sensor is located in an environment with very low or very high temperatures that are not withstood by standard silicon chips. Accordingly, an interconnecting cable is required between the sensor and the read-out circuit. However, this cable offers an equivalent series resistance (ESR) that behaves as a parasitic element for the measurement circuit. A typical value of that parasitic resistance is 0.3–0.4 Ω/m [4], but this depends on temperature. The use of the circuit in Figure 1a in such remote scenarios can be modeled as shown in Figure 1b, where *R*_w1_ and *R*_w2_ are the parasitic ESR of the two interconnecting wires. In these conditions, for a standard platinum Pt100 thermal sensor (with a nominal resistance of 100 Ω at 0 °C), each meter of wire causes an error of around 1 °C, which is completely unacceptable for most instrumentation applications.

In order to tackle the previous limitation, some resistive sensors are interconnected to the circuit via a three- or four-wire connection instead of the typical two-wire configuration, although these are more expensive solutions. In that sense, several interface circuits have been recently proposed to read three- [5,6] and four-wire [7] resistive sensors. An alternative is to keep the two-wire connection and incorporate some extra components at the sensor end, such as a couple of twin diodes [4,8,9,10,11,12], a Zener diode [13], a diode-controlled switch [14], or a capacitor [15]. The wireless read-out of resistive sensors is also feasible [16], but the distance between the sensor tag and the reader is usually limited to a few centimeters.

The main features of the circuits intended for two-wire resistive sensors based on a couple of twin diodes [4,8,9,10,11,12], which are the most popular in the literature, are summarized in Table 1. The circuits proposed in [4,8,9] have a simple topology, but they show a significant non-linearity error (NLE). These rely on the charging–discharging process of an RC circuit that is controlled by a microcontroller unit (MCU) and/or a 555-timer. A remarkable drawback of these circuits is the mismatch in the forward voltage of the two diodes, especially because these have a different forward current. Such a limitation can be solved using more complex topologies that provide a constant current excitation (CCE), as in [10,11,12]. The circuit in [10] had four analog switches at the input controlled by a digital clock, and four sample and hold (S&H) circuits at the output connected to an adder–subtracter amplifier. It showed very good linearity, with a maximum NLE of 0.026% Full-Scale Span (FSS). The circuit in [11] was based on a Howland current source with a square-wave excitation at the input. This provided a square-wave output signal that was averaged to extract the sensor resistance without the wire-resistance effects. The excitation current of the topology proposed in [11], however, clearly depends on the mismatch of the four resistors involved, and, in addition, the circuit can suffer from instability issues due to the positive feedback. The circuit in [12] was based on a bipolar three-step current source and the measurement of six output voltages. This circuit was able to estimate the forward voltage of the two diodes and, hence, compensate for any potential mismatch between them, but it was quite complex in terms of the circuitry required for both excitation and reading.

Taking into account the previous context, this paper proposes and experimentally characterizes a novel twin-diode-based front-end circuit for two-wire resistive sensors. The circuit has a very simple topology but a performance even better than that in [10], as summarized in the last row of Table 1 and explained in more detail in the following sections.

The paper is organized as follows. Section 2 explains the operating principle of the proposed circuit and its non-idealities. Section 3 describes the materials and methods applied to test the circuit under different scenarios. Section 4 reports and discusses the experimental results, especially in terms of the input–output characteristic. Finally, Section 5 draws the main conclusions and anticipates potential future research work on this direction.

## 2. New Front-End Circuit

### 2.1. Overall Description

The novel circuit for remote resistive sensors with a two-wire connection is shown in Figure 1c. With respect to the basic inverting-amplifier topology in Figure 1b, there are three main differences: (i) a square-wave excitation at the input, as in [11], (ii) the inclusion of two diodes (*D*_1_ and *D*_2_) in the feedback path, and (iii) the presence of a low-pass filter (LPF) at the output. Unlike the simple circuits proposed in [4,8,9], the circuit in Figure 1c forces the same forward current (which is also independent of the sensor resistance) through the two diodes, thus achieving better matching in their forward voltage.

The resistive sensor (*R*_x_) and the two diodes are located at a certain distance from the circuit and then connected via two wires that are modeled by *R*_w1_ and *R*_w2_. As represented in Figure 1c, two remote operating regions are distinguished [13]. Diodes are located in region A, whereas the sensor is in region B. For example, for a thermal application, only the sensor will be in direct contact with the object to be measured (i.e., region B), whereas the diodes will be as close as possible to the sensor but not in direct contact (i.e., region A). Region A encloses both diodes so that their operating temperature is expected to be the same. This can be reinforced by using a package that includes both diodes, which also ensures better matching in their current–voltage characteristic.

### 2.2. Operating Principle

The operating principle of the circuit in Figure 1c is as follows, assuming a bipolar excitation signal (*v*_in_) with a duty cycle of 50%. In the positive semicycle of the input signal, the current through *R*_ref_ is *I*_ref_ = *V*_ref_/*R*_ref_, where *V*_ref_ is the amplitude of the square input signal. This current then goes through *R*_w1_, *D*_1_, and *R*_w2_, thus generating a voltage at the OpAmp output equal to

(1) $$V_{N}=-\left[\frac{V_{\mathrm{ref}}}{R_{\mathrm{ref}}}\left(R_{w1}+R_{w2}\right)+V_{F1}\right]$$

where *V*_F1_ is the forward voltage of *D*_1_ at a forward current equal to *I*_ref_. On the other hand, in the negative semicycle of the input signal, we have *I*_ref_ = −*V*_ref_/*R*_ref_. This current circulates through *R*_w1_, *D*_2_, *R*_x_, and *R*_w2_, and, therefore, the OpAmp output voltage becomes

(2) $$V_{P}=\frac{V_{\mathrm{ref}}}{R_{\mathrm{ref}}}\left(R_{w1}+R_{x}+R_{w2}\right)+V_{F2}$$

where *V*_F2_ is the forward voltage of *D*_2_ at a forward current equal to *I*_ref_, which is the same (in absolute value) to that affecting *D*_1_ in the positive semicycle, and, hence, it is reasonable to assume that *V*_F1_ = *V*_F2_. Accordingly, the OpAmp output offers a bipolar square signal (*v*_o1_), shifted 180° with respect to the input, and with a positive (negative) amplitude of *V*_P_ (*V*_N_), as represented in Figure 1d.

The periodic signal resulting from the previous stage has an average value that can be computed by

(3) $$\bar{V_{o1}}=\frac{1}{T}\int_{0}^{T}v_{o1}\left(t\right)dt=\frac{1}{T}\left[\int_{0}^{T/2}V_{P}dt+\int_{T/2}^{T}V_{N}dt\right]=\frac{V_{P}+V_{N}}{2}$$

where *T* is the period of both *v*_in_ and *v*_o1_. Assuming *V*_F1_ = *V*_F2_ and then replacing (1) and (2) in (3) results in

(4) $$\bar{V_{o1}}=\frac{V_{\mathrm{ref}}}{2R_{\mathrm{ref}}}R_{x}$$

which is proportional to *R*_x_ and independent of *R*_w1_ and *R*_w2_.

In order to extract this average (or DC component), it is proposed to connect the OpAmp output to an LPF, as shown in Figure 1c. In such conditions, the output of the filter (*v*_o2_) is a DC voltage equal to $\bar{V_{o1}}$, as represented in Figure 1d. This DC signal can be then amplified and converted to digital, similarly to what is usually carried out in Figure 1a. Note that the topology proposed in Figure 1c behaves as a differential circuit that operates sequentially. On the one hand, the circuit acquires information about the sensor and the parasitic components thanks to *D*_2_. On the other hand, it acquires information only about the parasitic components thanks to *D*_1_. And, finally, it carries out the difference thanks to the ensuing LPF.

### 2.3. Operating Frequency

As for the frequency of the input signal, this has to be low enough to ensure that the low-to-high and high-to-low transitions (due to, for instance, the slew rate of the OpAmp) at the output are much shorter than the period of that signal. In addition, this frequency has to be high enough to facilitate the design of the LPF; note that a low-frequency signal would require an LPF with a very low cut-off frequency that involves very high values of resistance and capacitance. Accordingly, a frequency of units of kHz seems to be appropriate.

### 2.4. Non-Idealities

Under non-ideal conditions, the output of the circuit can undergo some errors, for instance, due to the mismatch of the two diodes, the input offset voltage (*V*_IO_), and the input bias current (*I*_IB_) of the OpAmp. As for the former, if we assume a mismatch Δ*V*_F_ = *V*_F2_ − *V*_F1_, the resulting average value at the output can be expressed as

(5) $$\bar{V_{o1,\Delta V_{F}}}=\frac{V_{\mathrm{ref}}}{2R_{\mathrm{ref}}}R_{x}+\frac{\Delta V_{F}}{2}$$

Comparing (4) and (5) shows that the mismatch in the diodes causes an offset error. A value of Δ*V*_F_ up to 2 mV was reported in [4,6,12]. However, it was higher (up to 5 mV) in [10] but lower (around 1 mV) in [11]; note that most of the previous values were obtained at a forward current of 1 mA, which is the same as applied here. In addition, this mismatch has been reported to be quite constant with temperature [10]. Assuming Δ*V*_F_ = 2 mV, from (5), the resulting offset error equals 1 mV, which corresponds to 1% FSS for the conditions set in the following experiments. However, as explained later in Section 3, the diodes selected herein showed better matching (to be precise, $\left|\Delta V_{F}\right|\;$= 0.2 mV). Therefore, from (5), the offset error due to Δ*V*_F_ is expected to be 0.1 mV, which corresponds to 0.1% FSS.

The analysis of the circuit assuming *V*_IO_ and considering *R*_ref_ >> (*R*_w1_ + *R*_w2_) results in the following approximated expression:

(6) $$\bar{V_{o1,V_{\mathrm{IO}}}}\approx \frac{\left(V_{\mathrm{ref}}+V_{\mathrm{IO}}\right)}{2R_{\mathrm{ref}}}R_{x}+V_{\mathrm{IO}}$$

According to (6), *V*_IO_ causes sensitivity and offset errors in the input–output characteristic. Considering *V*_IO_ = ±300 µV, which is the typical value of the OpAmp used later, the sensitivity error due to *V*_IO_ could be equal to ±0.03%.

As for the input bias current, the output can be affected by the current (*I*_IB-_) of the OpAmp inverting input but not by that of the non-inverting input since no resistance is connected to it. The analysis of the circuit assuming *I*_IB-_ results in the following expression of the average output voltage:

(7) $$\bar{V_{o1,I_{\mathrm{IB}-}}}=\frac{\left(\frac{V_{\mathrm{ref}}}{R_{\mathrm{ref}}}-I_{\mathrm{IB}-}\right)}{2}R_{x}-I_{\mathrm{IB}-}\left(R_{w1}+R_{w2}\right)$$

According to (7) and similarly to (6), *I*_IB-_ generates sensitivity and offset errors. However, considering *I*_IB-_ = ±1 pA, which is the typical value of the OpAmp used later, the sensitivity and offset errors obtained from (7) are much lower (at least, a factor of 10^5^) than those obtained from (6).

## 3. Materials and Methods

**Figure 2:**
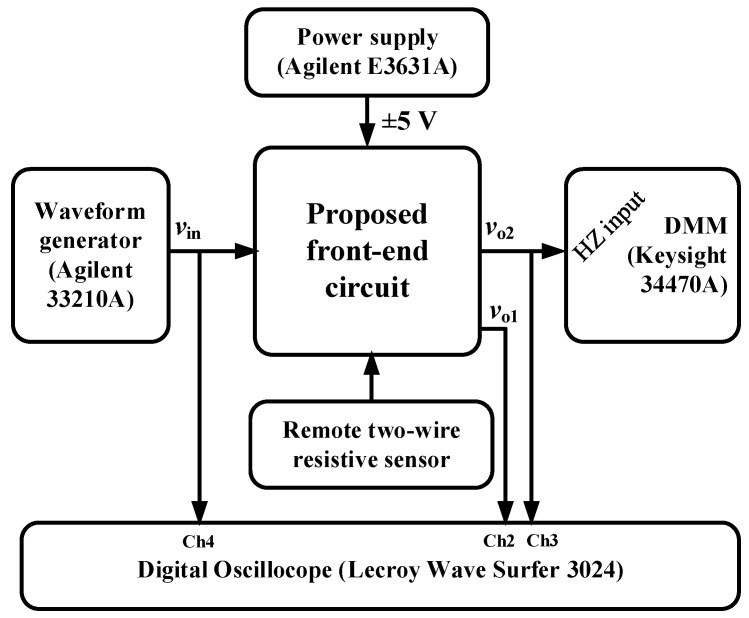
Experimental setup employed to test the circuit proposed in Figure 1c.

A prototype of the circuit shown in Figure 1c was built in a printed circuit board (PCB) using off-the-shelf components. Such a prototype was experimentally tested by means of the setup represented as a block diagram in Figure 2. The main features of the different blocks involved in this experimental setup are described in the following paragraphs. 

The OpAmp employed in the proposed front-end circuit was the TLC2274 (Texas Instruments) with a bipolar supply voltage of ±5 V, which was provided by a bench-top power source (Agilent E3631A). This OpAmp relies on an advanced CMOS (complementary metal oxide semiconductor) technology and offers a rail-to-rail output performance, high speed (with a slew rate of 3.6 V/µs), a low input offset voltage (with a typical value of 300 µV), and a very low input bias current (with a typical value of 1 pA). A waveform generator (Agilent 33210A) provided the input voltage (*v*_in_), which was a square signal with a frequency of 1 kHz, a duty cycle of 50%, and an amplitude (*V*_ref_) of ±1 V. The resistor *R*_ref_ in Figure 1c had a nominal value of 1 kΩ, and, hence, the resulting current through the resistive sensor was *I*_ref_ = ±1 mA, which is an appropriate value to have low self-heating effects in the sensor. 

The resistive sensor (*R*_x_) was emulated using resistors between 63 Ω and 267 Ω. Those resistors were of metal-film technology so as to have a stable response with respect to both time and temperature. The range of *R*_x_ selected corresponds to temperatures between −92 °C and +458 °C assuming that it emulates a Pt100 thermal sensor [17]. The parasitic resistance of the two interconnecting wires was also emulated by resistors, considering the three scenarios summarized in Table 2.Scenario #1 is the reference case with both wire resistances equal to zero, and, hence, there is no interconnecting cable between the sensor and the circuit.Scenario #2 involves two equal wire resistances that emulate an interconnecting cable with a length of around 10 m.Scenario #3 includes two wire resistances with a strong mismatch between them: one corresponds to an interconnecting cable with a length of 10 m, whereas the other corresponds to a cable with a length of 5 m if the same resistivity and section are considered. 

As for the diodes (*D*_1_ and *D*_2_) placed in the feedback path of the circuit shown in Figure 1c, we selected two general-purpose switching diodes (1N4148 from OnSemi). In order to have similar features (especially, in terms of forward voltage), the two diodes were chosen from the same batch. A preliminary experimental test of these two diodes showed that they had a very similar forward voltage (to be precise, *V*_F1_ = 609.9 mV and *V*_F2_ = 609.7 mV) at a forward current of 1 mA, which is the actual value employed in the circuit considering the value of the selected components. Such a measurement of the forward voltage was carried out at room temperature using a source and measurement unit (Agilent B2901).

The LPF represented in Figure 1c employed to extract the DC component was a simple passive first-order filter with a unity gain in the pass band and a cut-off frequency of 0.16 Hz. This filter was implemented with a resistor of 1 MΩ and a capacitor of 1 µF. For applications that require a fast transient response, it would be advisable to employ an active LPF of a higher order and with a higher value of the cut-off frequency. For example, an active LPF of second order with a cut-off frequency of 16 Hz would provide similar results at the output but with a faster transient response.

A 7 1/2-digit digital multimeter (DMM, Keysight 34470A) was employed to measure the actual value of the resistors and also the output voltage (*v*_o2_) of the circuit for the different conditions under test. In order to avoid the loading effects of the DMM on the circuit output, the DMM input was set in high-impedance (HZ) mode (i.e., higher than 10 GΩ). In addition, the measurement speed of the DMM was set with a Number of Power Line Cycles (NPLC) equal to 100 (which corresponds to an integration time of 2 s) so as to have a measurement result less sensitive to noise and interference. On the other hand, a four-channel digital oscilloscope (Lecroy Wave Surfer 3024) was employed to monitor the waveform of the voltage at the main nodes of the circuit proposed in Figure 1c. As represented in Figure 2, the waveform of *v*_in_, *v*_o1_, and *v*_o2_ was monitored by means of channels 4, 2, and 3, respectively, of the oscilloscope. The loading effects of the digital oscilloscope on the LFP output (*v*_o2_) were avoided by placing an intermediate OpAmp acting as a voltage follower.

**Figure 3:**
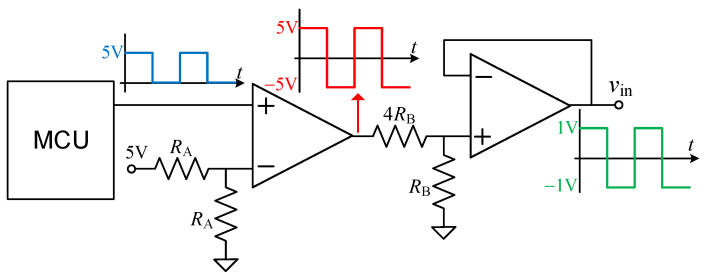
Circuit employed to generate the bipolar square signal applied to the input of the circuit shown in Figure 1c, with *R*_A_ = *R*_B_ = 100 kΩ.

The performance of the circuit in Figure 1c was also tested experimentally using a low-cost oscillator at the input, instead of the bench-top waveform generator. The circuit employed for that is shown in Figure 3. First, a low-cost MCU (ATtiny2313 from Microchip, operating at 5 V and 20 MHz) generates a unipolar square signal at a frequency of 1 kHz. This signal is applied to the non-inverting input of an OpAmp (TLC2274) acting as a comparator, with a bipolar supply voltage of ±5 V. As a result, at the output of the comparator we have a bipolar square signal of ±5 V at the same frequency. Then, the amplitude of that signal is reduced by 5 through a voltage divider. The resulting bipolar square signal of ±1 V is then provided at the output by means of a voltage follower.

## 4. Experimental Results and Discussion

### 4.1. Experimental Waveforms

**Figure 4:**
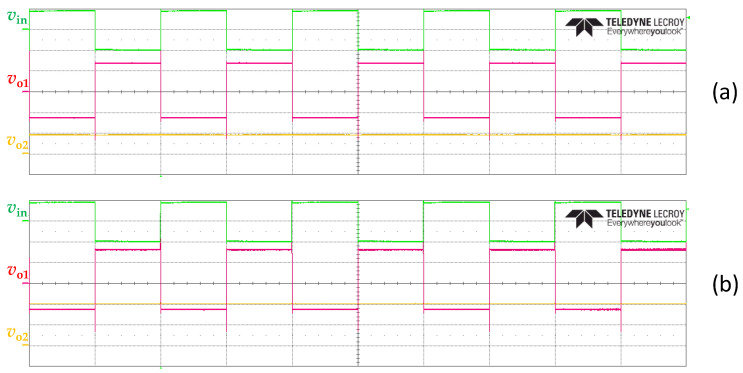
Experimental waveform of the voltage at the main nodes of the circuit proposed in Figure 1c for a sensor resistance with a nominal value of (**a**) 100 Ω and (**b**) 220 Ω. The 4-channel digital oscilloscope was set with a horizontal scale of 500 µs/div and a vertical scale of 500 mV/div for channel 2 (corresponding to *v*_o1_), 50 mV/div for channel 3 (corresponding to *v*_o2_), and 1 V/div for channel 4 (corresponding to *v*_in_).

Before extracting the input–output characteristic of the proposed circuit for the different scenarios under test, the waveform of the voltage at the main nodes of the circuit was experimentally monitored. Figure 4a,b show, for example, the waveform of *v*_in_, *v*_o1_, and *v*_o2_ when the sensor resistance had a nominal value of 100 Ω and 220 Ω, respectively, under scenario #2. First, note that the obtained experimental waveforms were very similar to those expected theoretically (see Figure 1d). As for the input signal (*v*_in_), which is represented in green in Figure 4a,b, this was exactly the same for both values of sensor resistance, as expected, with an amplitude of ±1 V and a frequency of 1 kHz. The output voltage (*v*_o1_) of the OpAmp, which is represented in red in Figure 4a,b, depended on the value of the sensor resistance. Although the amplitude of this square signal during the negative semicycle was very similar in Figure 4a,b, the amplitude during the positive semicycle did increase with increasing the sensor resistance. As a consequence of that, the output voltage (*v*_o2_) of the LPF, which is represented in orange in Figure 4a,b, became a DC voltage that also increased with the sensor resistance. It was around 46 mV in Figure 4a and 101 mV in Figure 4b.

### 4.2. Input–Output Characteristic

**Figure 5:**
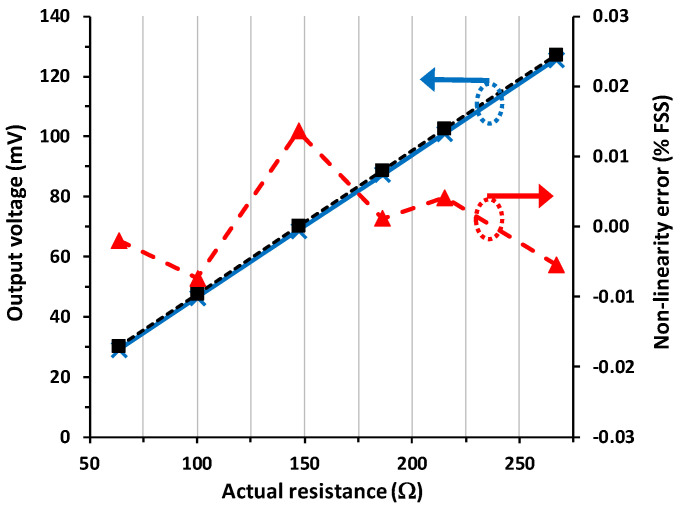
Theoretical (in black dashed line) and experimental (in blue continuous line) input–output characteristic of the circuit in Figure 1c for scenario #1 and the non-linearity error (in red dashed line) of the experimental response.

Figure 5 shows the theoretical (in a black dashed line and calculated by (4)) and experimental (in a blue continuous line) input–output (I/O) characteristic of the circuit in Figure 1c for scenario #1, which is the reference case with null parasitic resistances. The experimental response showed an offset error of around −1 mV, which can be ascribed to both Δ*V*_F_ and *V*_IO_, as inferred from (5) and (6), respectively. Figure 5 also shows the NLE, which was calculated by fitting a straight line to the experimental data using the least-squares method and then expressed as a percentage of the FSS. The maximum NLE was 0.013% FSS, which is a very remarkable value considering the simplicity of the proposed circuit. 

**Figure 6:**
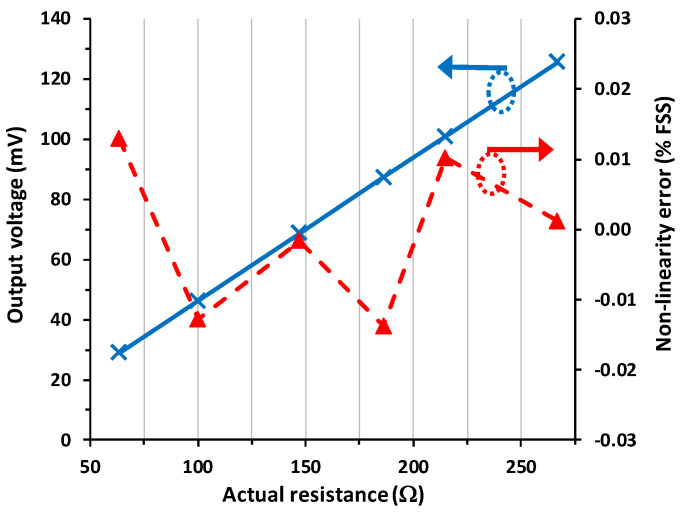
Experimental input–output characteristic (in blue continuous line) of the circuit in Figure 1c for scenario #2 and the corresponding non-linearity error (in red dashed line) expressed as a percentage of the FSS.

Figure 6 shows the experimental I/O characteristic and the resultant NLE for scenario #2, which corresponds to a 10 m interconnecting cable. The output voltage obtained in Figure 6 was very similar to that presented before in Figure 5. Actually, the maximum relative error with respect to scenario #1 was 0.03% FSS. Note that if the circuit in Figure 1b was employed in such conditions, the relative error due to the wires would be 4% FSS, which is more than a hundred times higher than that indicated before. In addition, the presence of the parasitic resistances did not affect the linearity of the circuit. The maximum NLE (in absolute value) in Figure 6 is 0.014% FSS, which is almost the same as obtained in Figure 5.

**Figure 7:**
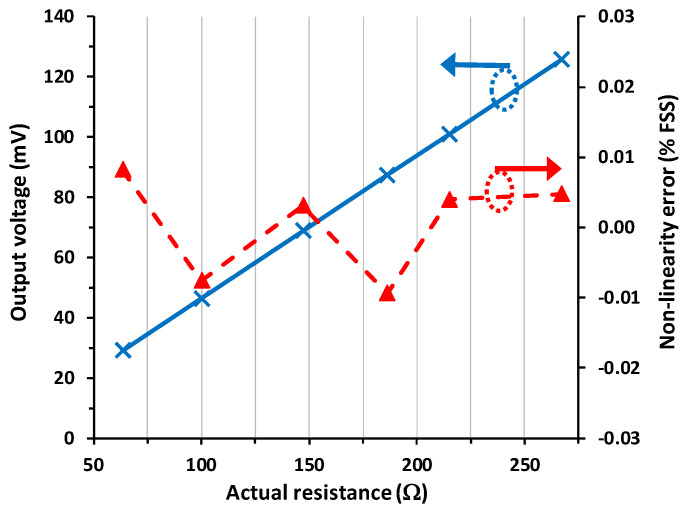
Experimental input–output characteristic (in blue continuous line) of the circuit in Figure 1c for scenario #3 and the corresponding non-linearity error (in red dashed line) expressed as a percentage of the FSS.

The I/O characteristic and the NLE for scenario #3, with an intended mismatch in the wire parasitic resistances, are represented in Figure 7. Despite the strong mismatch applied in the wire parasitic resistances, the resulting output voltage was almost identical to that obtained in Figure 5, with a maximum relative error with respect to scenario #1 of 0.01% FSS. The linearity was not either affected by the mismatch of the parasitic resistances. The maximum NLE (in absolute value) in Figure 7 is 0.009% FSS, even slightly lower than those presented before.

When the circuit in Figure 3 was employed instead of the bench-top waveform generator, the circuit in Figure 1c showed a very similar performance. For example, for scenario #1, the maximum NLE was 0.016% FSS instead of the 0.013% FSS obtained in Figure 5. Accordingly, a high performance can be obtained even using a low-cost oscillator at the input. The average current consumption, which was measured with the DMM indicated in Section 3, of the circuit in Figure 1c together with the oscillator in Figure 3 (but excluding the MCU) was 4.4 mA, which is comparable to that reported in [3]. This consumption could be reduced using a low-power OpAmp.

### 4.3. Discussion

According to Figure 5, Figure 6 and Figure 7, the circuit in Figure 1c shows an excellent linearity (around 0.01% FSS), and this is independent of the presence and also of the mismatch of the wire resistances. As stated in Table 1, the proposed circuit shows the best performance in terms of linearity. In comparison to the circuit suggested in [10], which is clearly a more complex solution, the NLE here is two times better. On the other hand, in comparison to the circuit proposed in [11], which has a similar operating principle but uses a current source with more limitations, the NLE here is more than 30 times better. Further, the output voltage itself in Figure 1c is also insensitive to both the wire resistances and their mismatch, with a relative error, with respect to the reference case, in the range of 0.01–0.03% FSS. This is at least two orders of magnitude lower than that obtained if the circuit in Figure 1b was employed instead.

## 5. Conclusions

A simple front-end circuit for remote two-wire resistive sensors was suggested. The circuit relies, on the one hand, on an inverting-amplifier topology that provides a constant current to the sensor and, on the other hand, on a couple of twin diodes at the sensor end. Despite the simplicity of the proposed circuit, its output signal is insensitive to the wire resistances, and this was proved theoretically and experimentally. According to the experimental results, the proposed circuit shows a remarkable linearity (around 0.01% FSS) and a very low relative error (in the range of 0.01–0.03% FSS with respect to the case with null parasitic resistance). As a future research work, two lines are considered: on the one hand, the implementation of the topology in Figure 1c in a low-power version so as to be better adapted to the field of autonomous sensors and, on the other hand, the applicability of the proposed topology to other types of sensors, such as remote resistive sensors with a three-wire connection.

## Figures and Tables

**Table 1:** Main features of circuits for two-wire connected resistive sensors using a couple of twin diodes.

Ref.	Circuit Topology	CCE	Range (Ω)	Max. NLE (% FSS)
[4]	MCU and 3 switches	No	[100, 146]	1.04 ^1^
[8]	555-timer in astable mode	No	[148, 826]	1.07 ^2^
[9]	MCU, 555-timer, and 2 switches	No	[100, 150]	0.33
[10]	4 switches, 4 S&H, and an amplifier	Yes	[49, 315]	0.026 ^2^
[11]	Howland current source with a square excitation	Yes	[100, 1300]	0.41 ^2^
[12]	Bipolar three-step current source	Yes	[100, 214]	Not available
This work	Inverting amplifier with a square excitation	Yes	[63, 267]	0.013

^1^ According to the comparative analysis reported in [9]. ^2^ Calculated a posteriori here using the experimental data reported in those references.

**Table 2:** Value of the resistors emulating the parasitic resistance of the wires for the three scenarios under test.

Scenario	*R*_w1_ (Ω)	*R*_w2_ (Ω)	Others
1	0	0	Reference scenario
2	3.9	3.9	Emulating a 10 m cable
3	3.9	2	Emulating a strong mismatch between the two wires

## Data Availability

The data that support the findings of this study are available upon reasonable request from the author.
